# Health services for women, children and adolescents in conflict affected settings: experience from North and South Kivu, Democratic Republic of Congo

**DOI:** 10.1186/s13031-020-00265-1

**Published:** 2020-05-27

**Authors:** Chiara Altare, Espoir Bwenge Malembaka, Maphie Tosha, Christopher Hook, Hamady Ba, Stéphane Muzindusi Bikoro, Thea Scognamiglio, Hannah Tappis, Jerome Pfaffmann, Ghislain Bisimwa Balaluka, Ties Boerma, Paul Spiegel

**Affiliations:** 1 Centre for Humanitarian Health, Department of International Health, Johns Hopkins Bloomberg School of Public Health, Baltimore, MD USA; 2 Ecole Régionale de Santé Publique, ERSP, Faculty of Medicine, Université catholique de Bukavu, Bukavu, Democratic Republic of Congo; 3 Foundation RamaLevina, Bukavu, Democratic Republic of Congo; 4 United Nations Children’s Fund, Bukavu, Democratic Republic of Congo; 5 United Nations Children’s Fund, New York, USA; 6 Department of Community Health Sciences, Rady Faculty of Health Sciences, University of Manitoba, Winnipeg, MB Canada

**Keywords:** Health services, health system, Conflict, Population displacement, North Kivu, South Kivu, Democratic Republic of Congo, Maternal, Newborn, Child, Reproductive health

## Abstract

**Background:**

Insecurity has characterized the Eastern regions of the Democratic Republic of Congo for decades. Providing health services to sustain women’s and children’s health during protracted conflict is challenging. This mixed-methods case study aimed to describe how reproductive, maternal, newborn, child, adolescent health and nutrition (RMNCAH+N) services have been offered in North and South Kivu since 2000 and how successful they were.

**Methods:**

We conducted a case study using a desk review of publicly available literature, secondary analysis of survey and health information system data, and primary qualitative interviews. The qualitative component provides insights on factors shaping RMNCAH+N design and implementation. We conducted 49 interviews with government officials, humanitarian agency staff and facility-based healthcare providers, and focus group discussions with community health workers in four health zones (Minova, Walungu, Ruanguba, Mweso). We applied framework analysis to investigate key themes across informants.

The quantitative component used secondary data from nationwide surveys and the national health facility information system to estimate coverage of RMNCAH+N interventions at provincial and sub-provincial level. The association between insecurity on service provision was examined with random effects generalized least square models using health facility data from South Kivu.

**Results:**

Coverage of selected preventive RMNCAH+N interventions seems high in North and South Kivu, often higher than the national level. Health facility data show a small negative association of insecurity and preventive service coverage within provinces. However, health outcomes are poorer in conflict-affected territories than in stable ones. The main challenges to service provisions identified by study respondents are the availability and retention of skilled personnel, the lack of basic materials and equipment as well as the insufficient financial resources to ensure health workers’ regular payment, medicaments’ availability and facilities’ running costs. Insecurity exacerbates pre-existing challenges, but do not seem to represent the main barrier to service provision in North and South Kivu.

**Conclusions:**

Provision of preventive schedulable RMNCAH+N services has continued during intermittent conflict in North and South Kivu. The prolonged effort by non-governmental organizations and UN agencies to respond to humanitarian needs was likely key in maintaining intervention coverage despite conflict. Health actors and communities appear to have adapted to changing levels and nature of insecurity and developed strategies to ensure preventive services are provided and accessed. However, emergency non-schedulable RMNCAH+N interventions do not appear to be readily accessible. Achieving the Sustainable Development Goals will require increased access to life-saving interventions, especially for newborn and pregnant women.

## Introduction

**Fig. 1:**
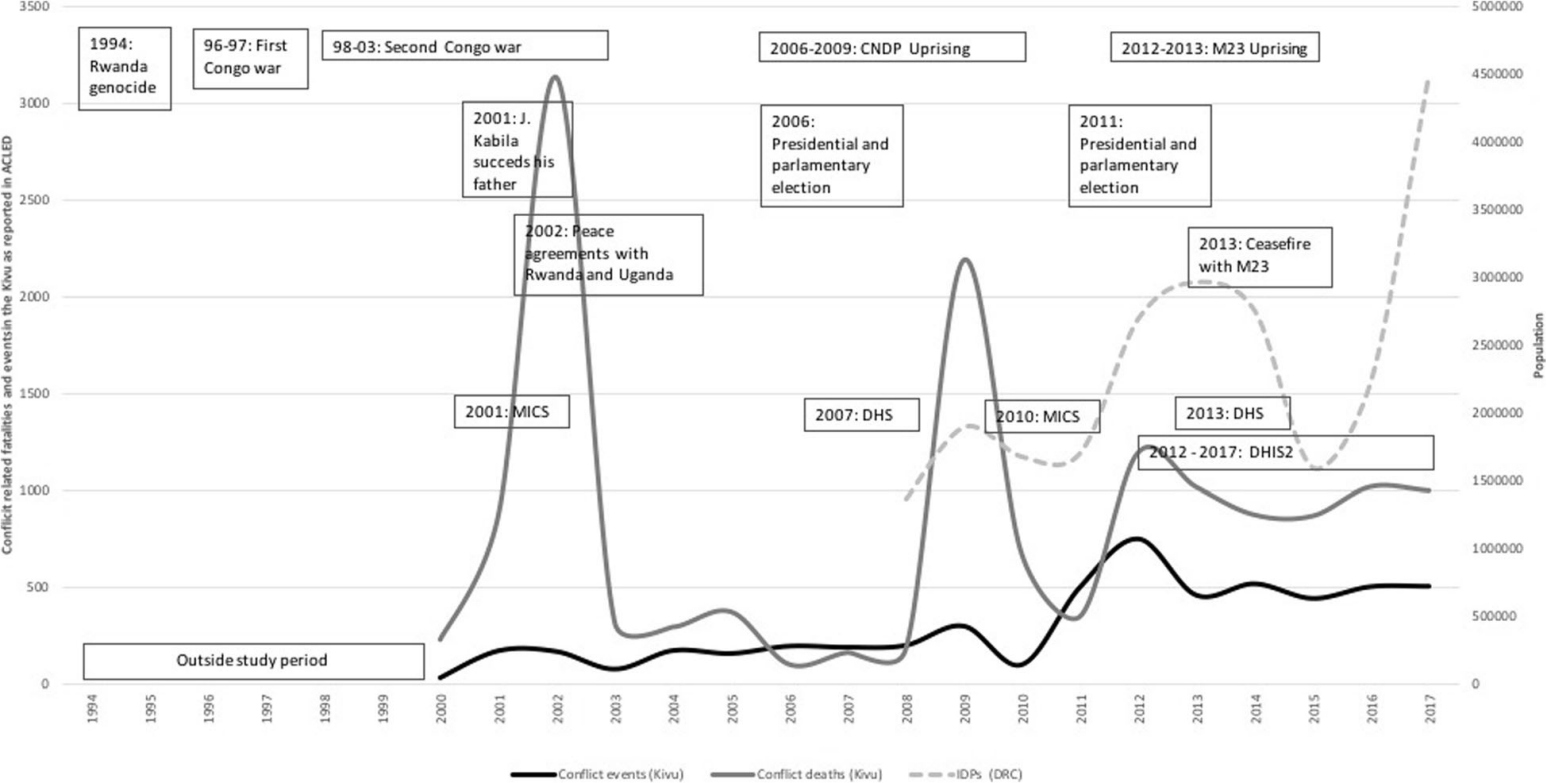
Timeline of key conflict events, intensity of violence and internal displacement in DRC and the Kivu provinces (1994–2017)

The Democratic Republic Congo (DRC) has experienced decades of violence that started in the early 1990s, and formally ceased in 2002 with a peace agreement signed by the warring parties (Fig. 1). While the peace led to a transitional government and elections in 2006, it did not end the fighting [1]. Pockets of insecurity persisted, especially in the eastern provinces of the country, mainly linked to the failing governmental institutions, perceptions of marginalization by the central government in Kinshasa, grievances over the allocation of resources, representation in the central government and service provision. Although the eastern region is no longer an “arena of muscular military intervention by its neighbors” [2], it saw a proliferation of small-scale armed groups along ethnic lines, entangled in unstable coalitions serving wider political and economic interests. Latest estimates counted 120 armed groups in North and South Kivu provinces, making the situation complex and difficult to control [2].

Humanitarian consequences are dire: almost one fourth of the two provinces’ population were classified as in need of humanitarian assistance in 2017 and 2018 (2.2 million people in 2017 and 1.6 million in 2018 in North Kivu; 1.4 and 1.7 million people in South Kivu respectively) [3, 4]. Recurrent clashes in North Kivu and resurgent confrontations in South Kivu have led to extensive population displacement (estimated 1.2 million and 650,000 internally displaced persons (IDPs), respectively [5]) and the declaration of the United Nation’s Level 3 emergency in October 2017, the highest as defined by the international humanitarian community. While overall mortality has decreased since the dramatic levels during the Congolese wars, maternal and child mortality remain high in DRC (846/100,000 live births and 104/ 1000 live births [6] respectively). Approximately half of the children under the age of 5 years are stunted, and preventable diseases are the main causes of child mortality in both provinces [6]. Little is known about adolescents’ health needs and few services exist. After being used as a war strategy, sexual violence has permeated the Congolese society and continues representing a major threat to women’s and men’s wellbeing. While the burden is difficult to estimate, 9000 new cases have been reported in 2017 just in North Kivu alone [7].

Health service delivery in such a context is challenging and influenced by a variety of factors. Little evidence exists about how health services for women and children are delivered in humanitarian settings and how contextual factors shape health system functioning. The objectives of our study were twofold: first, describe reproductive, maternal, neonatal, child, adolescent health and nutrition (RMNCAH+N) service provision in North and South Kivu; and second, investigate factors influencing decision making and service delivery.

This study is part of a multi-country study coordinated by the BRANCH (Bridging Research and Action in Conflict settings for the Health of Women and Children) Consortium and focused on RMNCAH+N in ten conflict-affected countries: Afghanistan, Colombia, DRC, Mali, Nigeria, Pakistan, Somalia, South Sudan, Syria, Yemen [8].

## Methodology

### Study design

We used a case study design, modified from a standardized protocol agreed upon by the BRANCH consortium and adapted for each country context. In DRC, we conducted a mixed-methods case study combining a desk review of publicly available peer-reviewed and grey literature, primary qualitative and secondary quantitative data. The qualitative component aimed to understand how contextual factors shape RMNCAH+N decision making and service provision. The quantitative component focused on estimating coverage of RMNCAH+N interventions at provincial and sub-provincial level, and according to exposure to conflict.

### Study setting

The case study focused on both North and South Kivu provinces in DRC, which have seen the start of the two Congolese wars and have undergone extensive violence since then. North Kivu has experienced higher intensity violence than South Kivu, both in terms of casualties and events. It was the location of 40% of all violent events occurring in DRC between 2012 and 2017 and suffered more casualties than all other DRC provinces until 2016 when violence erupted in Kasai. Few fatalities have occurred in South Kivu since 2012, despite numerous violent events throughout the years. Violence against civilians (33.2%) and battles with no change of territory (31.2%) were the most frequent forms of violent episodes in both provinces [9]. Variation in conflict-related events and deaths at the sub-provincial level is significant. In North Kivu, the territories of Rutshuru and Beni were the most affected in absolute numbers of casualties; Walikale becomes the most affected territory when considering population size, followed by Beni. In South Kivu, Uvira and Kalehe were the two most affected territories in absolute terms, and Uvira and Walungu in relative terms (see Additional file 1 for more details). (Note: from an administrative point of view, the DRC is organized in 26 provinces and 192 territories. The Health System follows a different structure: provinces are divided in health zones, and these in health areas.)

**Fig. 2:**
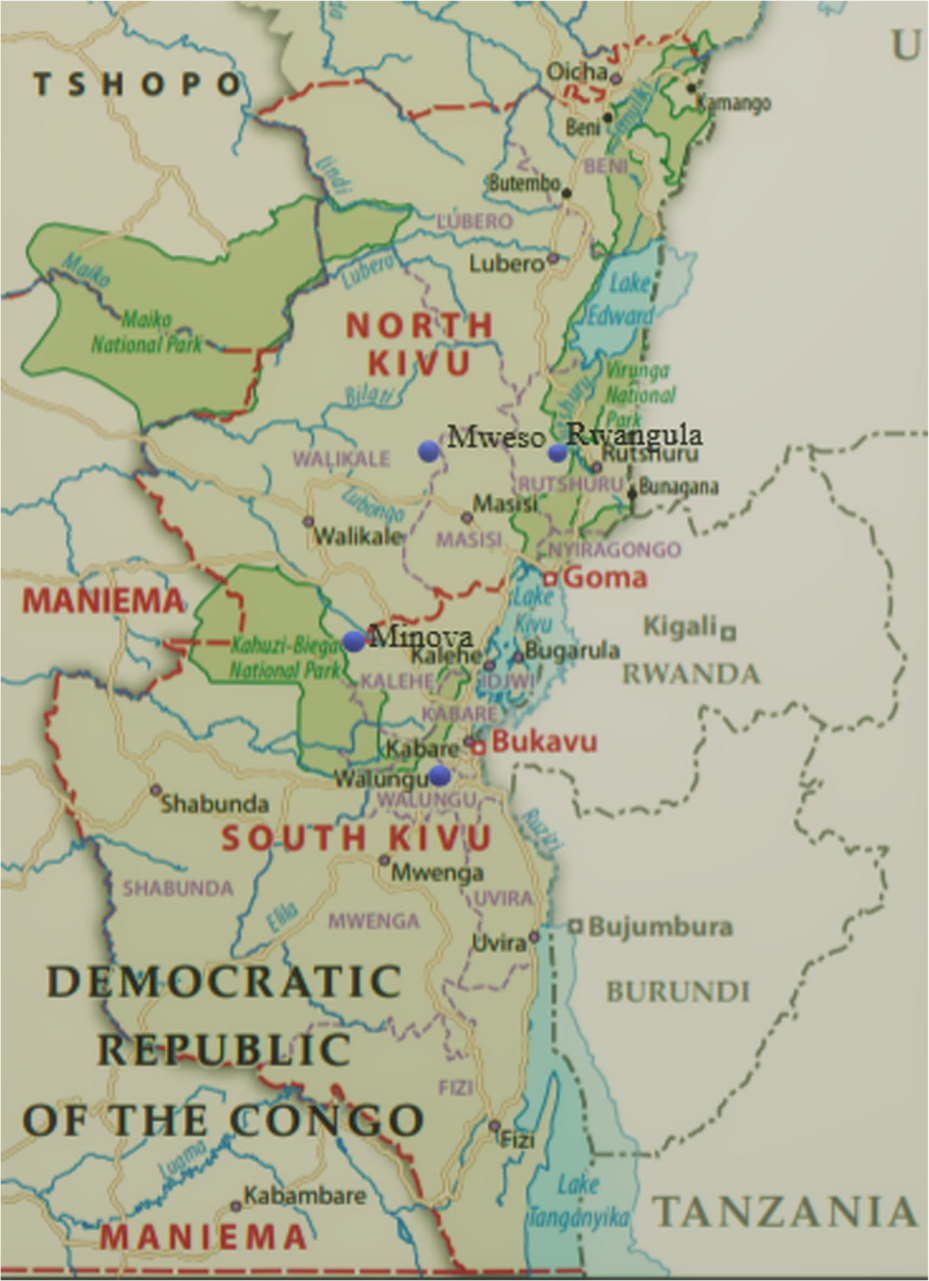
Map of North and South Kivu with qualitative case study sites (Mweso and Ruanguba in North Kivu; Minova and Walungu in South Kivu). (Adapted from [1])

The qualitative field work occurred in four health zones that were chosen for their history of conflict and insecurity (as per active armed clashes and population displacement) during the last 5 years together with accessibility. Selection was conducted with representatives of the Provincial Health Offices due to their knowledge of the provinces. In North Kivu, the research team visited the health zones of Mweso (Masisi territory) and Ruanguba (Rutshuru territory). Mweso experienced extensive violence that led to population displacement and attacks on health facilities, while Ruanguba was at the center of the March 23 Movement (M23) offensive in 2012–2013 [10]. In South Kivu, Minova (Kalehe territory) and Walungu (Walungu territory) were visited (Fig. 2); both were characterized by extensive conflict over land issues and customary power [11].

### Desk review

The investigators conducted a desk review of relevant grey and peer reviewed literature. Iterative searches were conducted in both Pubmed and Embase databases by combining key and MESH terms related to RMNCAH service delivery and North and South Kivu. We limited the search to the period 2000 to 2017. We did full text review of 71 results and of those 23 were relevant to our study. We also searched the grey literature for reports about health service provision in North and South Kivu using humanitarian websites such as Reliefweb, Humanitarian Response, Global Health and Nutrition Cluster. An additional 60 reports were reviewed that provided additional contextual information about the health system in DRC.

### Qualitative data

Qualitative data originated from individual or group interviews with representatives of private and public health service providers currently working in North and/or South Kivu (see details in Table 1). Fifty-one in-depth interviews (IDI) and four focus group discussions (FGDs), with a total of 84 respondents were conducted (Table 2).

**Table 1:** Additional information on qualitative and quantitative data used in the case study

**Informants for qualitative data** Individual and group in-depth interviews were conducted with: i) Ministry of Health officials from the Provincial Office in charge of RMNCAH+N programs, as well as the Chief Medical Officers responsible for the selected health zones; ii) Staff of United Nations agencies and of national and international non-governmental organizations including senior program managers, technical leads and other positions responsible for RMNCAH+N program planning, implementation and coordination; and. iii) Healthcare providers including clinicians in charge of RMNCAH+N services, chief nurses and community health workers. Participation was voluntary. Oral informed consent was obtained from all participants. Participants needed to be 18 years of age or older and working in the position for more than 30 days. **Quantitative data sources** Following data sources were used: i) The Armed Conflict Location and Event Data (ACLED) [12] provided data on type and number of conflict events, number of fatalities, location and date. This is a reliable conflict data source and one of the leading data sets in conflict epidemiology [12–14]. ii) The 2001 and 2010 Multiple Indicator Cluster Surveys and 2007 and 2013–2014 Demographic and Health Surveys (DHS) reports [6, 15, 16] provided data on key interventions coverage indicators in the RMNCH+N continuum of care at national and provincial levels. Coverage is expressed as the proportion of people who benefited from a certain service among the target population. iii) National Health Facility Information System as available in the District Health Information Software 2 (DHIS2). In South Kivu, health facility data existed for the period 2012–2017; in North Kivu only for the period 2015–2017. Facility data analyses were restricted to selected RMNCH indicators for which data availability allowed for comparisons and trends assessment (i.e. first visit of Antenatal Care (ANC1); fourth visit of antenatal care (ANC4), third dose diphtheria -pertussis -tetanus vaccine (DPT3), measles immunization and Caesarian section rate for South Kivu, and ANC1, ANC4, DPT1, DPT3, assisted deliveries, caesarean section, maternal mortality for North Kivu). Health facility data were merged with conflict events at territory level using Microsoft Excel to allow the assessment of the effects of conflict on RMNCAH indicators. iv) The 2017–2018 Service Provision Assessment report [14] provided information about the proportion of health facilities providing RMNCAH+N services. v) Population estimates from the provincial health divisions: the estimates are irregularly updated based on health-related activities such as distribution of insecticide-treated bed nets and were used to estimate intervention coverage based on the health facility data.

**Table 2:** Participants to in-depth interviews and Focus Group Discussions in the qualitative study component, North and South Kivu, DRC

Respondent by affiliation	North Kivu	South Kivu
Ministry of Health (DPS/MCZ)^a^	4	5
Non-governmental organization	4	6
United Nations agency	2	2
Health providers	12	12
Community Health Workers (focus groups discussions -FGD)	17	20
Total	39	45
Total participants	84	

Notes: ^a^*DPS* Division Provinciale de Santé (Provincial Health District), *MCZ* Médecin Chef de Zone (Chief Medical Officer of the health zone)

In each health zone, one hospital, one health center and one health post were visited. The chief nurse was interviewed in each facility. Two referral hospitals (one per province) were included. In each of them, we interviewed the head of the maternity department and the chief midwife. One FGD per health zone was conducted with community health workers (CHWs).

Relevant United Nations (UN) agencies, non-governmental organizations (NGOs) as well as national faith based organizations implementing RMNCAH+N programs were invited to participate to the study. We aimed to include five NGOs per province to ensure a variety of opinions was captured.

A four-day training was conducted in Bukavu to secure a common understanding of the study objectives and data collection tools. One interview guide for each targeted group was developed and tested in a health center in Bukavu that was not included in the sample. The interview guides for FGD and facility health workers were translated into Swahili. Data collection took place over 8 weeks from August to September 2018 and was typically completed over 3 days per health zone. Interviews in Bukavu and Goma were scheduled between field visits according to the availability of participants.

### Quantitative data

Multiple sources of secondary quantitative data were used to investigate coverage of RMNCAH+N interventions between 2000 and 2017. All were publicly available or were requested from the Ministry of Health (MoH). These included the 2001 and 2010 Multiple Indicator Cluster Surveys (MICS), 2007 and 2013–2014 Demographic and Health Surveys (DHS) [6, 15, 16] as well as routine health facility data available via the online District Health Information System (DHIS2) (details in Table 1). The Armed Conflict Location and Event Data (ACLED) project provided data on conflicts events and fatalities [9, 12].

### Definition of insecurity

We defined conflict intensity by territory as the average number of conflict-related fatalities divided by the average annual population between 2012 and 2017. As no standard threshold exists, we considered the distribution of deaths and classified territories with a conflict death rate over 20 per 100,000 persons per year as the most insecure (see Additional file 1). Using this definition on South Kivu, we classified as stable the territories of Idwji and Bukavu; as intermediate the territory of Kalehe^1^; and as conflict-affected the remaining territories of Shabunda, Fizi, Kabare, Mwenga, Uvira and Walungu. Of the seven territories of North Kivu, two (Beni and Walikale) were classified as insecure and the remaining five as stable.

### Outcome variables

Key indicators in the RMNCAH continuum of care were extracted from MICS and DHS reports and used for trends analysis at national and provincial level. They included the percentage of all women, currently married women and sexually active unmarried women aged 15–49 currently using any modern family planning method; skilled birth attendance; antenatal care (ANC); three doses of diphtheria, pertussis, and tetanus vaccine (DPT); measles vaccination; percentage of children under age 5 with symptoms of acute respiratory infection for or whom advice or treatment was sought from a health facility or provide; and oral rehydration therapy for children with diarrhoea. Stunting and wasting prevalence data were also extracted from MICS and DHS reports. At health zone level, RMNCAH indicators with sufficient data were derived from the DHIS2, namely first visit of Antenatal Care (ANC1); fourth visit of antenatal care (ANC4), third dose of diphtheria -pertussis -tetanus vaccine (DPT3), measles immunization and Caesarian section rate for South Kivu, and ANC1, ANC4, DPT1, DPT3, assisted deliveries, caesarean section, maternal mortality for North Kivu. As denominators, we used estimated populations from the health zones available from DHIS, given that the last DRC population census dates back to 1984. For interventions programming, the DRC ministry of health update the populations estimates by applying a population growth rate of 3%. Occasionally, population estimates are based on local censuses conducted prior to mosquito nets distribution campaigns. In South Kivu, for instance, the last population count was performed in 2014.

### Data management and analysis

Qualitative data were collected in French or Swahili depending on the preference of the respondent. Audio recordings in both languages were transcribed in French. Data were managed and coded in NVivo [17]. A combined approach to codebook development was used, with predefined codes addressing the specific issues the study aimed to explore, completed by additional codes arising from unexpected participants’ experience. Two team members coded the transcripts after having tested and compared coding approaches to ensure harmonization.

Framework analysis was used to explore qualitative data. A matrix output (with cases as row and codes as column) was developed to systematically summarize data and facilitate constant comparison within and across cases and topics [18].

With regard to quantitative data, we aggregated health facility data at territory level and merged them with the ACLED data, with Microsoft Excel, to enable analysis by conflict location. In fact, from an administrative point of view, the DRC is organized in 26 provinces and 192 territories, the latter being the lowest administrative unit in the ACLED. The Health System follows a different structure: provinces are divided in health zones (33 health zones in North Kivu, 34 in South Kivu, and 516 nationwide).

DHIS2 data from South Kivu were checked for completeness and errors, as they included raw numbers aggregated at the health zone level. It was therefore possible to asses reporting completeness as well as denominators used in calculations; this was not feasible for DHIS2 data from North Kivu as only proportions as estimated by the MoH in North Kivu were available. The reporting completeness for South Kivu ranged between 75% (in 2013) and 96% in 2017. Since we had no good empirical basis for adjustments for incomplete report, we assumed that all non-reporting facilities provided zero services (see Additional file 3 for more details).

The quantitative analysis comprised three steps: first, descriptive trends analysis was carried out to compare two coverage indicators (ANC1 and DPT3), one service provision indicator (caesarean section rate) and two institutional mortality indicators (maternal mortality and stillbirths), in conflict-affected versus more stable territories. The choice of these indicators was dependent on data availability in the DHIS2. Second, the Composite Coverage Index (CCI) [19] was calculated at the national and provincial level using DHS and MICS data for both North and South Kivu. The CCI is a weighted average coverage of eight preventive and curative interventions, namely oral rehydration therapy, acute respiratory infection, family planning, skilled birth attendance, antenatal care, measles vaccination and diphtheria, pertussis, and tetanus vaccine. The CCI gives equal weight to four stages of the RMNCAH continuum of care: family planning, maternal and newborn care, immunization, and case management of sick children, and has been shown to be a robust measure of RMNCH continuum of care with a strong association with health outcomes [20]. Third, the effect of insecurity on service provision in South Kivu was estimated by random-effects generalized least square regression modelling, with year as random effect to account for the temporal variability of the conflict intensity and numbers of violent episodes. Regression analysis was run on health facility (DHIS2) data aggregated at the territory level and linked with conflict events. We used Stata 15 [21] for this analysis and set the significance to *p* < 0.05. The paucity of data from North Kivu precluded the regression analysis for this province.

## Findings

### Availability of RMNCAH+N services in north and south Kivu

#### Common interventions and delivery modalities – qualitative results

Overall, health services in North and South Kivu are provided through an essential package (*Paquet minimum d’activités*) offered through primary health care facilities (health centers) and community health workers; and a complementary package (*Paquet complementaire d’activités*) provided at the hospital level. The minimum package includes family planning, antenatal- and postnatal care, obstetric care (for normal deliveries), newborn care, child vaccination, integrated management of childhood illness (IMCI), and treatment of severe acute malnutrition. The complementary package also covers internal medicine, hospitalization, intensive and emergency care (including emergency obstetric and newborn care), surgery, gynecology, as well as preventive and promotional activities, and managerial functions [22]. Table 3 provides more details about the level and delivery modalities of RMNCAH+N interventions along the continuum of care as reported by informants and as described in the literature. Complicated cases are referred to secondary or tertiary institutions, however rapidity and effectiveness of referral depend on the existence and functioning of a transport system.

**Table 3:** RMNCAH+N Interventions provided in North and South Kivu

Along the continuum of care, RMNCAH+N interventions are delivered at following levels in North and South Kivu: • Promotion of essential preventive practices related to hygiene, appropriate child feeding, malaria prevention, as well as some component of IMCI occur at community level; • Antenatal consultations, normal deliveries, postnatal care, vaccination and IMCI are provided at the health center level; • Family planning, treatment of sexually transmitted infections (STIs), and HIV testing are provided at hospital level. Family planning is also offered at health centers and health posts. However, it was reported that *“reproductive health is forgotten in post-crisis or emergency situations. Partners may bring nutrition and protection but forget that reproductive health is really an essential component both in normal times, and even more in conflict or post conflict situations”* (Provincial MoH). Attempts are made by provincial authorities to ensure that the Minimum Initial Service Package (MISP) is included in all emergency programs; • Comprehensive care for sexual assault survivors is usually provided at the hospital level, although post-exposure prophylaxis (PEP) kits are available also in many health centers. The multi-sectoral support package includes medical and psychosocial treatment, legal support and socio-economic reintegration; • Nutrition programs for the treatment of severe acute malnutrition are few and supplementary programs addressing moderate acute malnutrition almost inexistent (to be noted is that prevalence of wasting is usually below emergency level). “*UNICEF is practically the only [actor] who provides therapeutic products and Plumpy’Nut to malnourished children in this country*” (UN) and is confronted with important supply challenges; • Programs focusing specifically on adolescents are still very new and seldom implemented. Service providers recognize that “*we are as well adolescents in the implementation of these services […*] *but we notice that there is interest among adolescents who come as soon as we start providing this service*” (Health Zone Chief Medical Officer). Adolescents are usually treated together with the rest of the patients. The few health centers that have specific interventions for adolescents focusing on reproductive health and sexually transmitted infections are supported by external technical partners (e.g. NGOs with a specific mandate for this group); and • Given the community-based approach streamlined by the MoH, all health facilities organize and work through community-based actors (see Table 4).	

The implementation of the packages varies importantly in reality depending on the presence of a technical and financial partner (TFP) such as international NGOs or national religious organizations supporting the health facility with equipment, drugs and human resources. These factors led to a high heterogeneity among health areas and zones in terms of availability and quality of services. Overall, TFPs operate through a health system strengthening approach by supporting services provided in existing health facilities and community health services, even during times of instability. Mobile clinics are used by the majority of TFPs only when no health facility exists, and the population has no access to health services. Community outreach includes activities at health posts and treatment sites as well as CHWs (see Table 4 for more details).

**Table 4:** Community health services in North and South Kivu

**Community health services** • **Health posts**: Established when communities are more than five km away from the health center. They are run by a nurse responsible to treat a limited number of conditions for children and adults (e.g. malaria, diarrhea, acute respiratory tract infections). No inpatient service is available, and deliveries are usually not conducted; • **Treatment sites:** Established in areas where one or more villages have difficulties in accessing treatment. Treatment of cases of malaria, diarrhea, and pneumonia without complications are provided by two CHWs who are trained and supervised by the nurse of the responsible health center; and • **CHWs**: National policy foresees one CHW every 10 villages, which are organized under “*cellule d’animation communautaire*” for every 40 villages. CHWs are chosen by the villages under their responsibility. No specific training is needed to become a CHW, but literacy and numeracy are required. CHWs are meant to be trained and supervised by the health center. CHWs refer serious cases to the closest health facility, conduct behavior change communication and contribute to the community- based epidemiological surveillance.	

#### RMNCAH+N service availability – quantitative results

A nationwide survey about service availability was conducted in 2017–2018, with a sample size of 1412 health facilities. Of these, 1380 could be assessed. Out of all assessed health facilities, 94 and 62 were in North and South Kivu respectively [23]. Nationally, 2.2% of the health facilities could not be visited, but non-response rates were considerably higher in North and South Kivu (4.1 and 12.7% respectively), presumably due to insecurity. Table 5 presents a selection of available RMNCAH+N indicators from North and South Kivu, compared with the national average as shown in the report. Variation seemed great: availability of services like Vitamin A supplementation, family planning and STI screening appeared higher in North and South Kivu than the national average. Other services like growth monitoring or cesarean sections seemed much lower in the South than in North Kivu. As discussed below, the offer of health services is likely to be driven by the longstanding presence of technical and financial partners in these provinces due to the protracted conflict.

**Table 5:** Proportion of RMNCAH+ N services availability in health facilities in North and South Kivu, in 2018. Selection of indicators extracted from the 2017–2018 Service Provision Assessment [23]

Service provided	North Kivu	South Kivu	DRC
*Number of assessed health facilities*	*N = 94*	*N = 62*	*N = 1380*
Facility type			
*Hospitals (referral or tertiary)*^*a*^	32	45	35
*Referral health centers*^*a*^	23	2	16
*Hospital centers/clinics*^*a*^	22	18	10
*Health centers*	22	35	39
Child growth monitoring service	**90**	76	89
Routine vitamin A supplementation	**83**	**77**	68
Child immunization^b^	85	86	90
*DPT3*	**99**	60	91
*Oral polio vaccine*	**99**	60	86
*Measles vaccine*	86	**100**	90
*BCG*	**80**	21	76
*All basic child vaccines*	**79**	21	70
Integrated management of childhood illnesses	**100**	**100**	100
Modern methods of family planning	**68**	**87**	68
*Reversible FP method*^*3*^	**72**	**87**	69
*Male or female sterilization*	**22**	**33**	17
*Any FP method, including periodic abstinence*	**72**	**87**	69
HF offering ANC	95	90	96
*1–2 times/week*	22	**87**	54
*3–4 times/week*	**1**	0	1
*≥ 5 times/week*	**69**	0	26
*Tetanus vaccine during ANC visit*	**81**	**90**	75
*Including all components of PMTC*^*4*^	**43**	9	28
Deliveries	89	88	96
Caesarean section	**30**	18	26
Voluntary screening of sexually transmitted infections	**77**	**77**	56
Emergency transport service	17	**64**	19

Notes: ^*a*^Secondary level health facilities officially permitted to offer CS services (78% in North Kivu, 65% in South Kivu and 61% nationwide); ^b^Routine provision of DPT/Pentavalent/Polio/Measles vaccines; ^3^ Following are defined as reversible family planning (FP) methods: oral contraceptive (combined pills or progestin-only pills), injectables (combined or progestin only), implants, intrauterine devices, male condoms, female condoms, Fertility awareness methods or abstinence. ^4^ PMTCT: Prevention of Mother to Child Transmission

The data presented are %. Values in bold are higher than or equal to national average

Source: [23]

#### Coverage of RMNCAH+N interventions – quantitative results

**Fig. 3:**
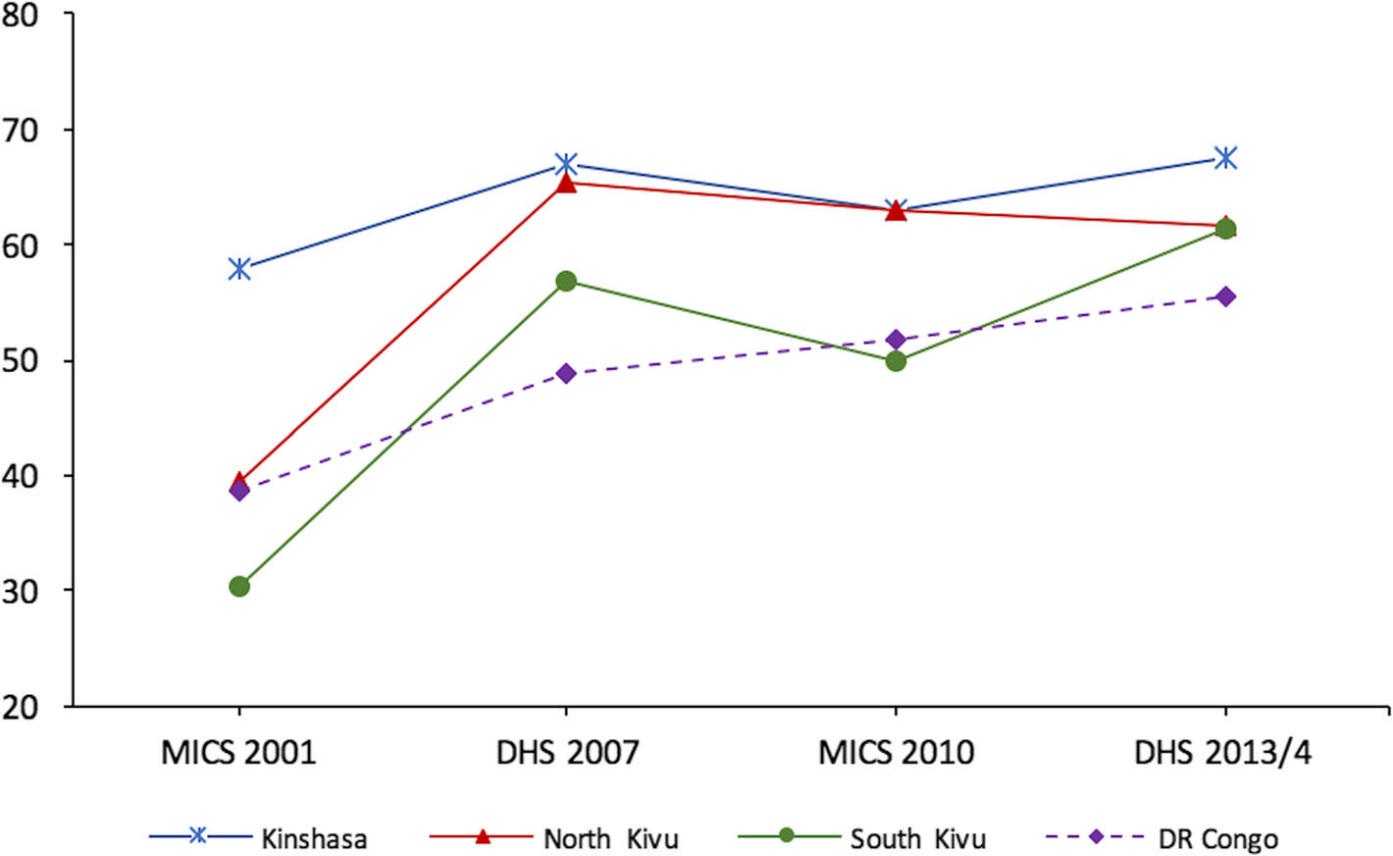
Trends in RMNCH composite coverage index in the Kivu and nationwide, household surveys, 2001–2014

Long-term trend analysis drew on two MICS (2000 and 2010) and two DHS (2007 and 2014) surveys. Almost all pre-selected clusters could be visited and the household and individual response rates in the Kivu’s were high. Overall, the RMNCH CCI increased in both provinces and nationwide during the years under study, going from 38.9 to 61.9% in North Kivu, from 29.2 to 63.9% in South and from 37.6 to 59.4% nationwide (Fig. 3 and Additional file 2). There is no evidence from the national surveys that the coverage trends in North and South Kivu were more severely affected by the conflict than other parts of DRC during 2000–2013.

**Fig. 4:**
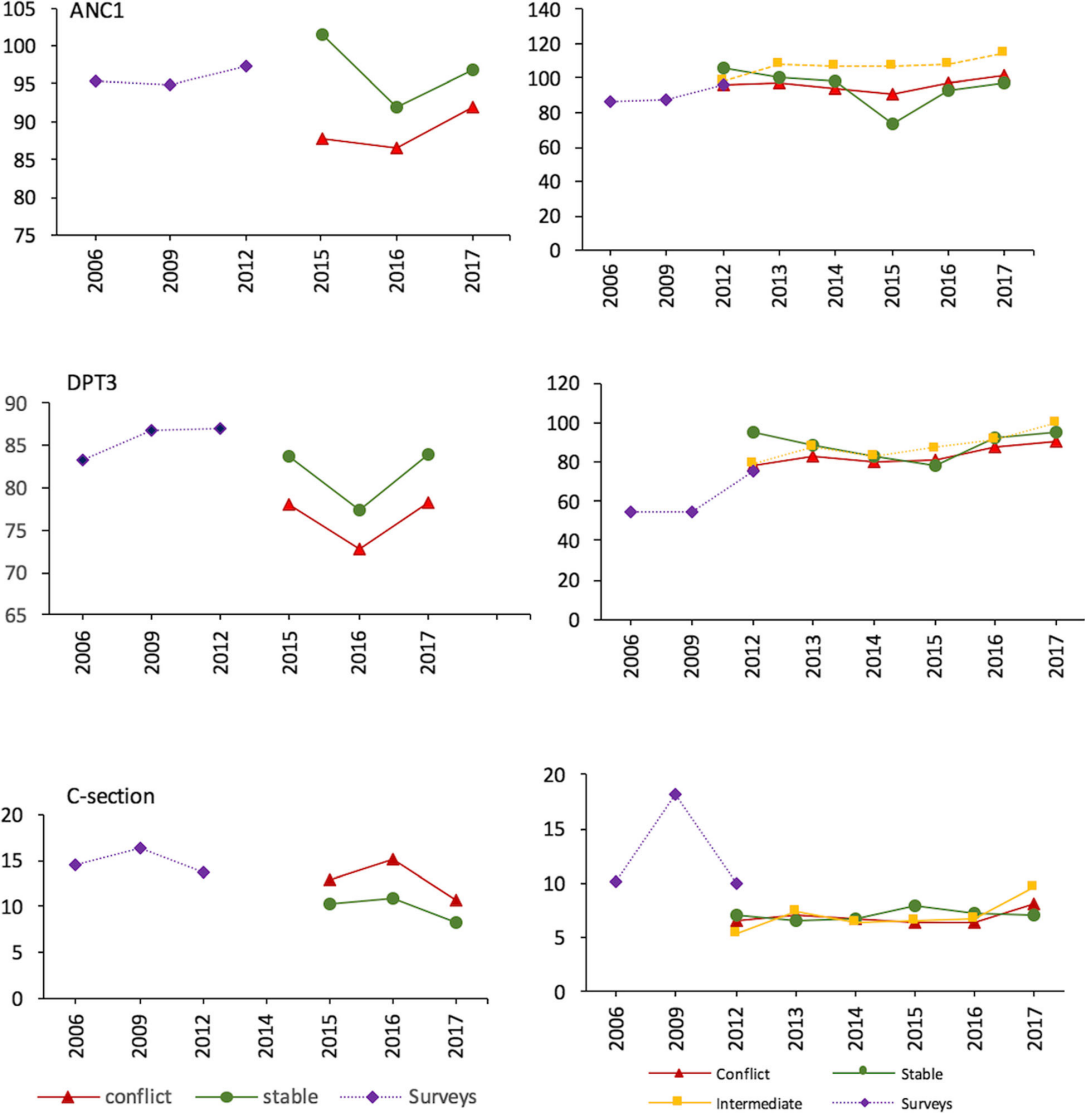
Trends in ANC1, DPT3 coverage and caesarian section rate; surveys and facility data for North (left) and South Kivu (right), 2006–2017

ANC1 coverage was high in the study provinces according to the DHS 2013 (97 and 96% in North and South Kivu respectively) and remained high during 2012–2017. According to health facility data, ANC1 coverage did not differ among the most and the least conflict-affected territories in South Kivu, whereas coverage appeared lower in conflict-affected territories between 2015 and 2017 in North Kivu (Fig. 4, panel A). The intermediate conflict exposure category (Kalehe territory in South Kivu) had a higher DPT1 coverage compared to stable territories. This territory received extensive population displacement (making population estimates less certain) and hosted an increasing number of national and international humanitarian actors that provided services [5, 24].

In the DHS 2013, DPT3 coverage was higher than national average (60%) in both North Kivu (87%) and South Kivu (76%). According to the health facility data, conflict-affected territories displayed lower coverage than stable ones in North Kivu but not in South Kivu (Fig. 4b). In North Kivu, measles immunization coverage in conflict territories was lower than in more secure ones and tended to decline over time (Additional file 2).

Caesarean rates in 2013 in both North (13%) and South Kivu (10%) were higher than the national average (5.1%) [6]. Caesarean rate in conflict territories was higher than in more secure ones in North Kivu, but no difference could be seen in South Kivu (Fig. 4c). The regression analysis for South Kivu, however, showed a statistically significant decrease in caesarean rate (coefficient: -1.61, 95% CI: − 2.78 to − 0.44, *p* = 0.007) in conflict territories compared to stable ones, when controlling for population size, annual number of conflict events, reporting completeness rate and year (Table 6). No effect of conflict intensity was found (Table 7).

**Table 6:** Regression results of the effect of conflict location on selected RMNCH indicators, South Kivu

Indicator	Stable	Intermediate		Conflict	
		Coefficient (95% CI)	*P* value	Coefficient (95% CI)	*P* value
ANC1	Ref.	16.92 (− 4.71 to 38.56)	0.125	3.92 (−19.10 to 26.95)	0.739
DPT1	Ref.	12.37 (3.69 to 21.07)	**0.005**	−0.18 (−11.97 to 9.61)	0.831
DPT3	Ref.	7.49 (−1.68 to 16.66)	0.109	−4.71 (−16.29 to 6.80)	0.423
Institutional Deliveries	Ref.	10.89 (−1.51 to 23.27)	0.085	−0.41 (−12.96 to 12.13)	0.948
Cesarean section	Ref.	−0.08 (− 0.1 to 0.83)	0.860	−1.61 (−2.78 to − 0.44)	**0.007**
Institutional stillbirth rate	Ref.	0.34 (0.11 to 0.58)	**0.004**	0.29 (−0.08 to 0.66)	0.122
Institutional Maternal Mortality Ratio	Ref.	1.71 (−30.19 to 33.61)	0.916	32.12 (−4.17 to 68.40)	0.083

Ref.: reference category. Conflict location variable has 3 categories corresponding to least insecure (stable), intermediate and conflict territories. Estimates were adjusted for population size, annual number of conflict events, reporting completeness rate and the year

**Table 7:** Regression results of the effect of conflict intensity on RMNCH coverage indicators and outcomes, South Kivu

Indicator	Coefficient (95 CI)	*P* value
ANC1	−0.19 (− 0.89 to 0.51)	0.597
DPT1	−0.08 (− 0.38 to 0.23)	0.614
DPT3	−0.18 (− 0.43 to 0.08)	0.183
Deliveries	−0.31 (− 0.76 to − 0.13)	0.166
C-section rate	− 0.02 (− 0.07 to 0.05)	0.642
Stillbirths	0.02 (0.01 to 0.02)	**< 0.001**
Maternal deaths	1.42 (0.64 to 2.20)	**< 0.001**

Estimates controlled for population size, year and reporting completeness rate

**Fig. 5:**
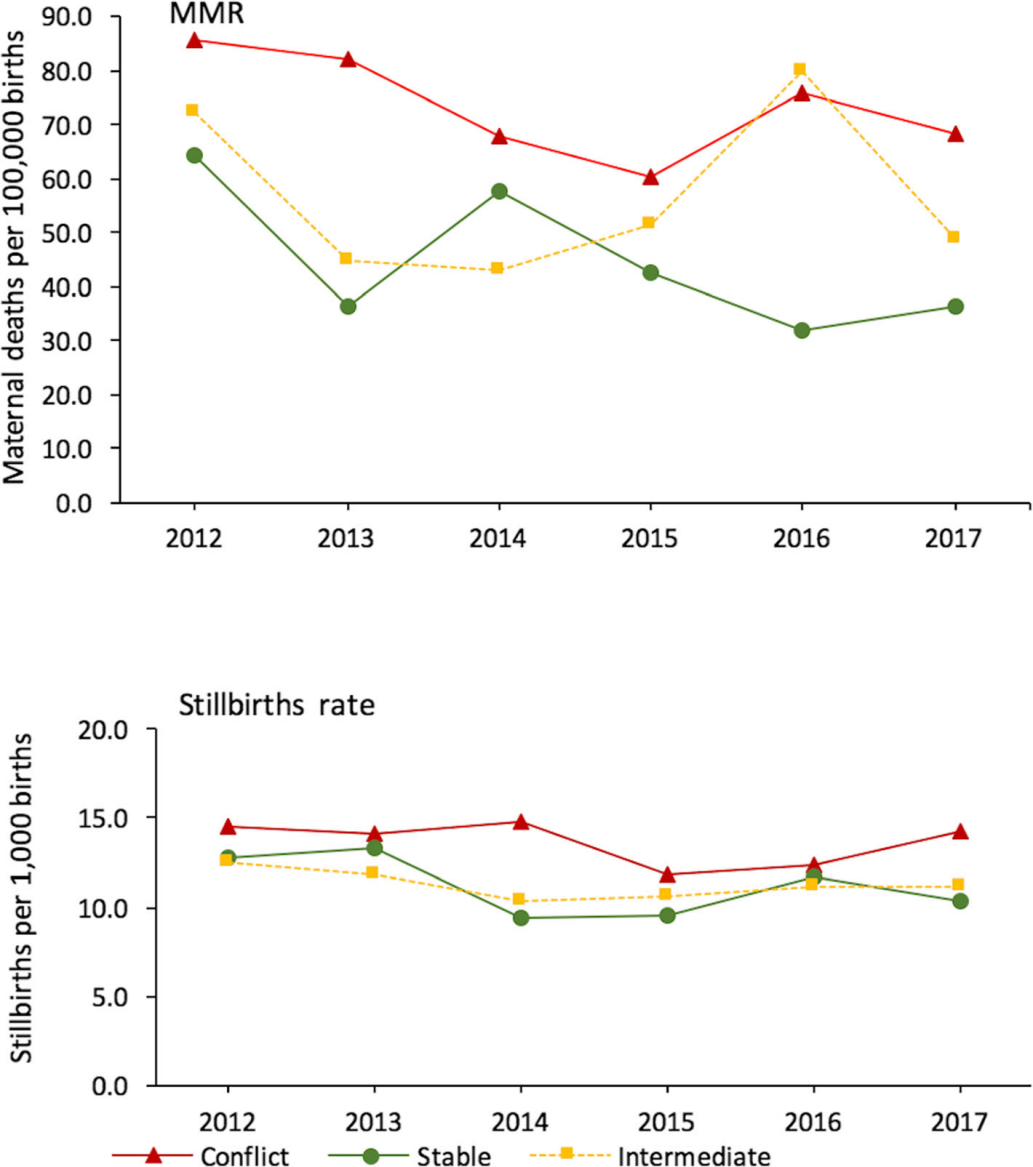
Trends in institutional maternal mortality ratio and stillbirth rate by conflict location, South Kivu, 2012–2017

In terms of health outcomes, 52 and 53% of children under the age of five are stunted in North and South Kivu respectively and stunting prevalence has been constantly higher in these provinces than the national average between 2001 and 2013–14; the gap seemed even widening (Additional file 2). Trend analysis of health facility data showed that conflict territories in South Kivu consistently reported a higher institutional maternal mortality ratio than stable territories and a higher stillbirth rate than the intermediate territory (Fig. 5). Estimates from the regression analysis were consistent with an incremental effect of conflict intensity on institutional maternal mortality (coefficient: 1.42, 95% CI: 0.64 to 2.20, *p* < 0.001) and stillbirth rates (coefficient: 0.02, 95% CI: 0.01 to 0.02, p < 0.001) (Table 7).

### Health system and contextual factors influencing decision making and service delivery in north and south Kivu

#### Insecurity

Insecurity is used as a decision criterion by NGOs for selecting intervention areas, as their humanitarian programs are meant to target conflict-affected populations. This is not the case for the government, which “*strives to provide services everywhere*” (PHD, North Kivu).

The current security context in the Kivu is characterized by moments of violence alternating with periods of calm. Activities are put on hold for a certain period of time and re-started once access is granted. This results in activities being delayed, but not necessarily canceled.

*“There are certain health facilities where sometimes you see that this month it’s calm, and you can go to the field and do your activities; the next month, they tell you it’s a red zone, and now you can’t go there”.* (NGO)

*“We have activities on a certain timetable. With this timetable, we say that on this day, this thing will be done. But when there is insecurity, we might be unable to get a certain thing done, so we postpone it. Instead of ending our program, we just prolong it until a different time”.* (Health worker)

Armed groups’ dynamics and strategies are complex, and driven by a variety of factors, ranging from grievance, need to affirm power, access to resources, etc. Study informants suggested that armed groups tend to protect the villages they originate from (or control) and allow for health activities to occur, yet at times after extorting illegal taxes from health providers. Attacks and plundering (or threat thereof) tend to target health facilities in zones controlled by adverse groups, although unpredictability is high.

*“When we vaccinate, everything stops, except breathing and heartbeats. [ …*] *even in the red health zones [ …*] *when we vaccinate, the armed groups calm down because they too have children and wives, and they allow vaccination to come in”* (NGO).

*“There is some collaboration with the armed groups to facilitate the transport of medications, because they themselves benefit from it” (Health worker).*

NGOs thus rely on dialogue with communities to strengthen acceptance and enable the safe implementation of activities.

*“For security, yes, the overall strategy that we use is to promote acceptance which is our trademark in our communities, and we ensure [ … ] that our programs are [of] high quality; that the promises we make to our beneficiary population are kept; we provide information first to the population and to the partners; for each commitment, we sign a protocol and we make every effort to respect this protocol; [ … ] we have codes of conduct for the staff, for our partners ensuring that we don’t do anything foolish in the community, codes of conduct linked to sexual life, to protection, against exploitation and sexual abuse, code of conduct against fraud, fraud sensitivity, and all that, because there are stories like that which once started create problems down the road”* (NGO).

Informants reported that operationally, insecurity affects service delivery through various channels: lack of human resources due to reduced access and fear of attacks; difficult working conditions affecting quality of care; lack of equipment and inputs due to pillaging; reduced investments and difficulties in procurement; and reduced revenues from patient visits (user fees apply in DRC – see below), as demand is limited to most severe cases.

Increased task delegation is thus often observed, both by shifting tasks to lower levels (e.g. replacing supervision by NGO and provincial health officers with “self-supervision” by the chief nurse) and to communities (e.g. diseases treatment, diagnostic and needs assessment are conducted by CHWs).

*“We trained the chief nurses from each health center on supervision; each one is considered as an internal supervisor and if the Health Zone Central Office team doesn’t arrive, the chief nurse acts officially as internal supervisor. Secondly, we trained the supervisors from the central offices of the health zone, so when the team from [NGO] is prevented from being there, the supervisors from the health zone do the supervision. Therefore, we have strengthened the system in such a way that if we cannot be there or are prevented from being there, we know they can still do something to ensure quality services”* (NGO).

#### Population displacement

UN agencies monitor population displacement and send out alerts highlighting areas with the highest humanitarian needs. NGOs then intervene to support displaced populations and hosting communities mainly through the “Rapid Response to Population Displacement” (*Reponse Rapide aux Mouvements de populations (*RRMP) [25]). This mechanism facilitates quick and coordinated response due to a pre-established partnership between the UN Children’s Fund (UNICEF) and a consortium of NGOs with predefined roles and responsibilities. RRMP interventions are usually limited to 3 months; hence, lack of continuity and sustainability of service provision are important issues.

Without external support, health facilities seemed to have little surge capacity to respond to population influx: health centers tend to be quickly overwhelmed, medication stocks run out and routine services lose effectiveness as resources are shifted towards the needs of the IDPs. Finally, displacement makes planning and procurement difficult.

*“With this insecurity [ …*] *a pregnant woman who should be taken care for in health zone A [ …*], *she comes to B. We have planned according to the population that we have, and now we have to care for one additional woman. When she arrives at B, she needs everything she would have gotten at A, and that affects our indicators, because we don’t know our denominator. A partner funds us according to the population we have but doesn’t factor in the surplus population: so when this new population arrives, we find that we don’t have sufficient supplies to care for them whereas we had planned according to our needs”* (Health Worker).

#### Leadership, governance and coordination

The DRC health system has a decentralized pyramidal structure organized in three levels: the central level is responsible for general sector policies, national priorities and tertiary hospitals; provincial offices provide technical support, administrative management and implementation supervision; health zones operationalize the national health plan, define, monitor and evaluate activities for primary health care, and interact with operational partners. Both public and private actors ensure service delivery. The private sector includes faith based and non-profit civil society (representing around 40% of the offer [23]) and for-profit service providers, this latter at all levels (from traditional healers, private pharmacies to clinics).

With support of the Global Financing Facility, DRC has established the Single Contract (“*Contrat Unique*”) in 2015 to reduce funding fragmentation and increase partner alignment in the implementation of the national RMNCAH+N strategy [26]. However, coordination and complementarity are not always achieved as donors and international actors often have their own priorities, causing some dissatisfaction among national authorities: “*If you are not part of our planning, you should go elsewhere*. *You cannot come in and impose. All work must conform with our vision. If it does not fit, we will tell them that their place is not here: perhaps elsewhere”* (Health Zone Chief Medical officer)*.*

In practice, given the extensive needs in the provinces and the limited public funding available, the government has little leverage to impose its plan of action and has to accept external priorities as *“the hand that gives is above the hand that receives”* (Health Zone Chief Medical Officer).

A parallel coordination and decision-making mechanism led by United Nations Office of the Coordination of Humanitarian Affairs (UNOCHA) and the health cluster is in place for emergency response. Here, UN agencies assess emergency needs and coordinate partners’ response. While the provincial health office is part of this mechanism, they are not leading this process and feel less involved in decisions.

#### Health financing

Public domestic resources for health corresponded to 1.14% of the Gross Domestic Product in 2014 (compared to 2.4% in Sub-Saharan Africa) and represented the same year 14% of the total expenditure for health at national level [26]. Limited funding is therefore available to ensure minimum infrastructure and equipment (e.g. water, electricity or beds). The main sources of funding are user fees and external aid. Patients cover the entire costs for consultation and medications, except for few cases when particularly vulnerable groups (e.g. displaced population or indigent households) are exempted by external actors subsidizing care. Attempts to implement voluntary health insurance systems (either community based or for specific population groups like state employees) have occurred since 2005, but have major sustainability problems and low coverage [27].

This results in a two-tiered system in which health facilities supported by external partners can provide more and better services than those relying mainly on out-of-pocket expenditure.

*“Our health zone evolves at “two speed”: in the health areas that are supported by a partner, there care is free as the partner pays for incentives, equipment, drugs [ … ] and the community utilizes these facilities. But the centers that are not supported and live on self-funding are almost not utilized [ … ] the population is poor [ … ] people prefer seek for care where facilities are supported … ”* (Health Zone Chief Medical Officer).

Performance-based financing (PBF) mechanisms also support service provision through bonuses to health facilities for the services they provide, taking both quantity and quality into account. Coverage is, however, limited, with 15 (out of 33) and 5 (out of 34) health zones employing PBF in North and South Kivu, respectively [28].

#### Health workforce

Lack of regular payment for health workers in non-supported facilities was identified as the greatest barrier to service provision by study participants. Salaries are low and rarely paid, making health workers dependent on incoming user fees. Health providers do not see their effort recognized and demotivation is high, impacting the quality of care provided. Turn-over is also high as health workers try to find better remunerated jobs in NGOs or at the provincial level. Insecurity also acts as a deterrent, causing numerous gaps in human resources in areas with the highest needs. Health facilities resort to “rapidly trained agents” to fill positions at the front line, although these lack formal training. Administrative strikes (e.g. only treatment services are provided) are recurrent, endangering disease surveillance and monitoring of activities.

#### Health service delivery

Physical access is a major challenge in DRC, where distance and road conditions restrict the access to certain areas. Insecurity and the rainy season exacerbate the situation. Certain locations are accessible only by motorbike or helicopter, constraining what can be really delivered (for logistical or financial reasons). Availability of essential medicament is further hampered by the existence of only one certified distribution center, with frequent stockouts. A referral transport system rarely exists especially in North Kivu (Table 5), and its effectiveness is reduced by road conditions.

Various efforts are in place to bring services closer to the communities: treatment and service are provided through CHWs; home visits are organized; treatment sites are set up; and mobile clinics are sent by NGOs.

### Strategies to overcome challenges in RMNCAH+N service provision

Organizations interviewed for the qualitative component reported examples of RMNCAH+N services that are particularly challenging in their implementation, as well as examples of operational adaptations to respond to these bottlenecks (Table 8). As evaluating the effectiveness of such efforts is beyond the scope of this study, the examples provided below simply describe organizations’ experience, without any assessment of relative effectiveness or cost-effectiveness.

**Table 8:** Examples of RMNCAH+N services that proved particularly challenging to be implemented in North and South Kivu, as reported by study informants

Factors	Challenging service	Efforts to overcome the challenge
Socio-cultural factors	• Family Planning • Abortion and post-abortion care • Care for survivors of sexual violence • Child health (services beyond vaccination such as growth monitoring or regular checks for early detection of conditions)	• Increase demand through awareness activities and build trust in the provider • Using family planning counselling as entry point for other sexual, reproductive or maternal services • Engage with difference audiences (youth, men, women, religious groups, midwives, teachers, etc) and work with champions • In communication messages, focus on the health risks for women and children (for example: the four “Too”: too many/ too early/ too close/ too late pregnancies) • Free care or input distribution
Health Service Delivery		
Lack of equipment/ ambulance	• Neonatal resuscitation • Referral	• Strengthen providers skills for early detection of complications • Anticipate delivery complications by admitting pregnant women weeks before due date in maternity waiting homes (*binyola)*
*Lack of trained human resources*	• Mental health for GBV victims • Maternal mortality surveillance	• Strengthen providers’ skills
Essential Medicines	• STIs, HIV • Undernutrition • General child and maternal health	• Improving procurement capacity to prevent stockouts • International procurement (by INGOs) • Resort to local pharmacies
Emergency context	• Adolescent health • Reproductive health	• Ensuring that MISP is included in all emergency programs

## Discussion

### Availability of RMNCAH+N services in north and south Kivu

The delivery of RMNCAH+N interventions continues in North and South Kivu, despite the numerous challenges and barriers that characterize the complex and unstable environment in Eastern DRC. In a context of chronic conflict with intermittent periods of violence, RMNCAH+N service provision is not completely hampered, but rather often temporarily delayed as it requires additional efforts by service providers in terms of access negotiation and logistics. Health facilities seem remaining open during times of insecurity for the most part, but often with fewer staff and medications available.

The effect of conflict on service provision is not straightforward to estimate. At provincial level, coverage of basic preventive schedulable child and maternal health services tend to be relatively high in North and South Kivu, and appears generally better than in many other non-conflict affected provinces in DRC. This is possibly due to the higher number of TFPs providing health services in these provinces than in the western part of the country since the Congolese wars [29]. In addition, a higher proportion of secondary or higher-level facilities were included in the nationwide assessment of service provision [23] compared to other provinces: this selection bias may partly account for higher coverage of selected procedures in North Kivu.

Within provinces, the overall impact of insecurity on preventive services may seem modest. We did not find evidence of differences in several intervention coverage between conflict and non-conflict affected territories in South Kivu. Coverage estimates from conflict affected territories in North Kivu appear lower than in non-conflict affected ones, however the paucity of data did not allow for statistical assessment. The prolonged effort by NGOs and UN to respond to humanitarian needs likely plays a major role in maintaining intervention coverage despite conflict. Task shifting and contingency plans enable TFPs to continue their activities although with delays. Allocating tasks to lower-level health care providers and CHWs are proving an effective strategy to increase access to service not only in resource poor countries [30], but also in humanitarian settings [31–33] and during major epidemics [34]. A major challenge of such a decentralized effort identified in crisis settings [32, 34], but applicable to the Kivu as well, is ensuring continuous access to essential medicines to CHWs, as supply chains often struggle to reach health centers. Mechanisms to anticipate stock-out and facilitate procurement should be foreseen.

Furthermore, as insecurity in the Kivus has become more and more driven by local armed groups and less by external actors, the consequences on health interventions are less predictable. Local armed actors often allow health actors to operate as a way to legitimate their power in front of the communities. Some might understand the importance of, for example, vaccinating the children of their own villages. Others may use it a source of extortion. Populations seem to have also learned how to adapt to insecurity in order to maintain some minimum access to health services. Study informants suggested that affected communities seek health services in neighboring zones, or adjust their health seeking behavior to insecurity by, for example, visiting the health facilities only when they see the NGO’s car approaching.

However, conflict does seem to have more serious consequences on health outcomes. While wasting was only sporadically reported above emergency level in small scale surveys publicly available online [35, 36], stunting affects half of the children under the age of 5 years in North and South Kivu, with prevalence higher than elsewhere in DRC. Recurrent outbreaks of preventable diseases such as measles and cholera also still continue to occur. This seems indicating that while sustaining the coverage of essential RMNCH services is critical during conflict, it does not suffice, as both the greater exposure to environmental hazards such as poor water quality and sanitary facilities, nutritional deficiencies and poor quality of care may contribute to elevated morbidity and mortality risks in conflict affected populations.

Furthermore, the higher maternal mortality and stillbirth rate in conflict-affected territories in South Kivu are likely to reflect delayed access to health care as well as lower quality care compared to the non-conflict affected territories. These results seem indicating that as opposed to schedulable preventive interventions, acute cases requiring life-saving interventions cannot be delayed. In such cases, even a temporary service interruption can have fatal consequences. Efforts to anticipate maternal and child emergencies are used (for example the “*binyola*”, e.g. a maternity waiting home where pregnant women are admitted a few weeks before due date to ensure access to the health care in case of insecurity or absence of emergency transport). Yet, these attempts cannot compensate for a steady access to functioning health facilities. Investments to ensure continuous more specialized expertise at health center level and a functioning referral system are necessary.

Interventions that appear lagging behind, such as family planning, post-abortion care, adolescent awareness raising and reproductive health services, are likely held back by cultural barriers on the demand side, the government reticence to invest in those interventions that are not considered a priority, or by the discrepancy between policy frameworks at the central level and operationalization at the provincial level. For example, family planning has experienced an increased relevance in the political agenda since 2012 with explicit ministerial support nationally and internationally, leading to the approval of the National Multisectoral Strategic Plan for Family Planning [37]. However, only 40% of all health zones nationwide offer family planning services and few partners support family planning in many provinces of the country [38]. In North and South Kivu, service availability is quite high according to the latest evaluation in 2017 (Table 2 [23]). Yet, utilization (as reported in the latest DHS) remains low [6], likely because of both financial barriers and cultural reasons. Organizations working in North and South Kivu are using different approaches to reach potential users and increase demand, such as the Balanced Counseling Strategy Plus [39]. Another example is the use of champions like religious leaders or well-known personalities to increase awareness about the topic. However, to achieve the stated goal of 19% modern contraceptive coverage, additional funding will be required [38].

Although the DRC has ratified the Maputo protocol, abortion is in reality nearly only performed when the mother’s life is at risk. Women wishing to terminate a pregnancy need to resort to unsafe methods, increasing the risk for adverse outcomes [40, 41]. However, last year’s publication of the Maputo protocol in the national legal gazette paves the way for increased legal access to abortion for all women in DRC [42].

Adolescent fertility rate in the DRC is among the highest in Sub-Saharan Africa [43]. At the same time, contraceptive use in the same age group is among the lowest [43], pointing to a real need for age specific interventions for adolescents. Despite gaining momentum, adolescent reproductive health and rights do not seem to be recognised among the most urgent health needs in DRC. Few TFPs have age-specific approaches and programs. A recent survey in South Kivu [44] reported low level of sexuality knowledge, but higher knowledge of contraceptive methods among adolescents. However, uptake remained low.

### Health system factors influencing decision making and service delivery in north and south Kivu

The reliance on external partners has been reported as the main challenge to a sustainable and nationally owned health service delivery in the Kivu. Despite its presence and the regulatory framework it provides, the state does not have the means to offer comprehensive health services. The population’s perception appears to be that the state has outsourced health service provision to technical partners. The existence of a parallel coordination system for acute emergency response, as well as conflicting policies (e.g. free service versus user fees) further exacerbates the fragmentation of the system, hinders real health system strengthening and service integration. What remains to be clarified is to what extent TFPs are simultaneously supporting and disempowering local authorities in their service provision role.

Another implication of the limited role of the state in RMNCAH+N service provision that study informants reported is the population’s mistrust in the government’s ability to protect its own citizens. This in turn shapes the way in which populations interact with national and international actors. Risk communication and community engagement represent major components of community health system strengthening activities aiming to support social accountability, service delivery, demand creation, and disease surveillance [45]. Lack of trust in the response has characterized the current Ebola epidemic in North Kivu, highlighting the need for increased community engagement and stronger role of national actors (religious and traditional leaders, civil society groups, youth and women associations, teachers, etc) to ensure effectiveness of the response [46–48].

With regard to health financing, the emergency approach of free care is difficult to integrate with the national policy of user fees for cost recovery. Particularly challenging is reintroducing user fees after a period of free care. In a country with 64% of the population under the poverty line, user fees represent an important barrier to health service utilization. Their removal, combined with other interventions to improve quality of care, is likely to have some short term benefits [49]. At the same time, as user fees are a major source of funding for health workers’ incentives, schemes proposing their abolition need to be implemented with caution to avoid further informalization of health care [50].

Human resources quality, management and retention (due to both health system and contextual factors) are major challenges for service delivery in the Kivus. Low and delayed salary payments force health workers to rely on multiple income sources as salary tends to suffice only for medical doctors [50]. Furthermore, fragmentation of income affects the time spent at the health facility and acts as a demotivating factor [50], reducing quality of services. Minimal opportunities exist in the public sector for training and professional development to update medical knowledge and practice. Lack of trained staff represents a major barrier for service provision and quality in facilities not supported by TFPs [51]. Staff continuously look for better professional opportunities leading to high turn-over and gaps. Second best solutions like the utilization of rapidly trained agents, who become long term instead of emergency solutions are often implemented despite their limitations.

Workforce capacity together with equipment availability and overall working conditions strongly affect quality of provided services, compromising the effective coverage of RMNCAH+N interventions provided in the Kivu.

### Limitations

This study has several limitations. First, one of the originally targeted health zones in North Kivu (Beni) was not accessible at the time of qualitative data collection due to the ongoing Ebola epidemic. Another health zone was therefore selected among the most affected by violent events.

Second, quantitative data from health facilities in North Kivu were not available for the entire study period, limiting the analysis we could conduct. Third, we relied on data from surveys and health facilities to establish the trends in coverage. While survey samples tend to avoid insecure areas, this did not seem a major issue in DRC. In fact, in the 2001 MICS, 32 out of 365 (9%) clusters across DRC were not accessible due to insecurity or change in the name of the site; they were replaced by nearby clusters. The report does not indicate where the clusters were and therefore it is impossible to know whether the study provinces were affected and how. In the 2013/2014 only 1 out of 32 clusters in North Kivu was not accessible. Reporting completeness could only be examined for South Kivu and could meaningfully impact the results if for instance it was much lower in conflict territories compared to intermediate and stable ones, particularly in North Kivu.

The health facility reports may also be biased and often have variable quality, and the population denominators for the coverage estimates have considerable uncertainty. On the positive side, however, the consistency between the coverage statistics based on the survey and annual health facility data did suggest reasonable quality. Fourth, ACLED data are aggregated at the territory level, while health facilities are aggregated at zone level. While ACLED data are geocoded, coordinates of the health zones are not available, preventing us to estimate the level of insecurity at the zone level. Additionally, our definition of conflict may not fully measure indirect effects and other dimensions of conflict through violence expressed in the form of destruction of property, abductions of children and women, sexual violence, illegal taxation, political repression, kidnappings, etc. [52]. Efforts to develop more multidimensional conflict exposure measures are encouraged in future studies*.* Finally, we only focused on the supply side given the broad scope of the study in terms of spectrum of services. As beneficiaries or patients were not interviewed, the study did not address the lack of demand as a possible cause of low coverage of RMNCAH+N programs.

## Conclusion

Health actors and affected communities have developed strategies to maintain the delivery of preventive schedulable RMNCAH+N services during the protracted and intermittent conflict that characterizes North and South Kivu provinces in DRC. Some RMNCAH indicators in the Kivus appeared to perform better than in other provinces of the country where there is not conflict, primarily due to external UN and NGO support with donor funding. Conflict-affected communities seem to have adapted their health care seeking behavior to ensure a minimum access to health services.

However, health outcomes appear to be poor and worse off in conflict-affected health zones in South Kivu, likely because non-schedulable acute RMNCAH+N interventions that aim to treat morbidity and save lives are not readily accessible to the population due to insecurity, shortage of trained human resources and funding. Achieving the Sustainable Development Goals will require increased access to life-saving interventions, especially for newborn and pregnant women.

## Supplementary information

**Additional file 1.** Conflict events and violence related mortality in North and South Kivu.

**Additional file 2.** Coverage estimates of selected RMNCAH+N interventions at provincial level, and related Composite Coverage Index.

**Additional file 3.** Assessment of health facility data quality at health zone and provincial level (North and South Kivu).

## Data Availability

Qualitative data is available from co-investigators upon reasonable request. Quantitative data is either publicly available or can be requested directly from respective Ministries of Health.

## Abbreviations

ACLED: Armed Conflict and Location Event Dataset
ANC1: First visit of Antenatal Care
ANC4: Fourth visit of antenatal care
BCG: Bacille Calmette-Guerin
BRANCH: Bridging Research and Action in Conflict settings for the Health of Women and Children
CCI: Composite Coverage Index
CHW: Community health worker
DHIS2: District Health Information System 2
DHS: Demographic and Health Survey
DPT1: First dose diphtheria -pertussis -tetanus vaccine
DPT3: Third dose diphtheria -pertussis -tetanus vaccine
DRC: Democratic Republic of the Congo
FGD: Focus Group Discussion
FP: Family Planning
HIV: Human Immunodeficiency Virus
IDI: In-Depth Interview
IDP: Internally displaced person
IMCI: Integrated management of childhood illnesses
MCZ: *Médecin Chef de Zone* (Chief Medical Officer)
MICS: Multiple Indicators Cluster Survey
MISP: Minimum initial service package
MoH: Ministry of Health
NGO: Non-governmental organization
PBF: Performance-based financing
PEP: Post-exposure prophylaxis
PMTCT: Prevention of Mother to Child Transmission
RMNCAH+N: Reproductive, Maternal, Newborn, Child and Adolescent Health plus Nutrition
RRMP: Rapid Response to Population Displacement
STI: Sexually transmitted infections
TFP: Technical and Financial Partners
UN: United Nations
UNICEF: United Nations’ Children Fund
UNOCHA: United Nations Office for the Coordination of Humanitarian Affairs

## Footnotes

1 Kalehe territory had a conflict fatality rate < 20/100,000 persons, but given its high number of internal displacements, it was regarded as an intermediate territory.
