# Supplementary material for: Health services for women, children and adolescents in conflict affected settings: experience from North and South Kivu, Democratic Republic of Congo
Source: Confl Health. 2020 May 27;14:31. doi: 10.1186/s13031-020-00265-1 (PMC7254646; doi:10.1186/s13031-020-00265-1)
Supplement: Supplementary file 1 — Additional file 1. Conflict events and violence related mortality in North and South Kivu. [file 13031_2020_265_MOESM1_ESM.docx]

Additional file 1: Conflict events and violence related mortality in North and South Kivu

Data source: ACLED [1]

Table 1: Number (and Proportion) of Conflict Events and Deaths in DRC, North and South Kivu, 2012-2017

| Conflict variables | 2012 | 2013 | 2014 | 2015 | 2016 | 2017 | Total |
| --- | --- | --- | --- | --- | --- | --- | --- |
| North Kivu |  |  |  |  |  |  |  |
| Events | 561 (54.0) | 362 (43.4) | 369 (35.0) | 336 (40.6) | 380 (39.3) | 335 (30.5) | 2343 (40.3) |
| Fatalities | 707 (48.7) | 939 (47.5) | 674 (54.5) | 773 (44.2) | 942 (54.1) | 795 (23.0) | 4830 (41.6) |
| South Kivu |  |  |  |  |  |  |  |
| Events | 193 (18.6) | 100 (12.0) | 152 (14.4) | 111 (13.4) | 126 (13.0) | 173 (15.8) | 855 (14.7) |
| Fatalities | 491 (33.8) | 82 (4.1) | 204 (16.5) | 112 (6.4) | 112 (6.4) | 208 (6.0) | 1209 (10.4) |
| National |  |  |  |  |  |  |  |
| Events | 1038 (17.8) | 834 (14.3) | 1053 (18.1) | 827 (14.2) | 969 (16.7) | 1098 (18.9) | 5819 (100) |
| Fatalities | 1453 (12.5) | 1977 (17.0) | 1237 (10.7) | 1749 (15.1) | 1741 (15.0) | 3452 (39.7) | 11609 (100) |

Data are n (%). Row percentages are presented for North and South Kivu with national numbers as denominators.

Table 2: Violent Events disaggregated by Type between January 2012 and June 2017 (North Kivu, South Kivu and National Level)

| Events types | North Kivu | South Kivu | National |
| --- | --- | --- | --- |
| Battle-Government regains territory | 136 (5.8) | 63 (7.4) | 248 (4.3) |
| Battle-No change of territory | 852 (36.4) | 332 (38.8) | 1817 (31.2) |
| Battle-Non-state actor overtakes territory | 79 (3.4) | 13 (1.5) | 116 (2.0) |
| Headquarters or base established | 37 (1.6) | 9 (1.1) | 65 (1.1) |
| Non-violent transfer of territory | 103 (4.4) | 24 (2.8) | 151 (2.6) |
| Remote violence | 43 (1.8) | 7 (0.8) | 54 (0.9) |
| Riots/Protests | 228 (9.7) | 102 (11.9) | 997 (17.1) |
| Strategic development | 159 (6.8) | 67 (7.8) | 441 (7.6) |
| Violence against civilians | 706 (30.1) | 238 (27.8) | 1930 (33.2) |

Figures 1 and 2 show the number of violence related fatalities and the number of violent events between 2012 and 2018, respectively. North Kivu has experienced higher intensity in violence than South Kivu, both in terms of casualties and events. North Kivu alone suffered more casualties than all other DRC provinces until 2016. This was no longer the case in 2017 due to the insurgence in violence in Kasai. Few fatalities have occurred in south Kivu since 2012 despite the occurrence of violent events throughout the years.

Figure 1: Number of violence related fatalities in north, south and in the other 9 drc provinces between 2012 and 2018. Source: ACLED

Figure 2: Number of violent events in North Kivu, South Kivu and in the other 9 DRC provinces between 2012 and 2017. Source: ACLED

Variation in conflict-related events and deaths at the sub provincial level is important.

In North Kivu, the estimated provincial population was 8,861,797 people in 2017 (Figure 3). The province has 7 administrative units (territories) organized in 33 health zones. Lubero and Beni territories have the highest numbers of health zones (9 and 7 respectively) while Nyiragongo has only one health zone.
Based on the ACLED data for 2012-2017, the annual absolute number of deaths ranged from 33 in Nyiragongo to 291 in Beni territory. Two territories (Walikale and Beni) had conflict intensity higher than 20 deaths per 100,000 population per year and were deemed most insecure (Figure 3). Apart from Nyiragongo with an annual conflict death rate of 17.3 per 100,000, all the other territories had a conflict death rate lower than 8.4. In fact, Nyiragongo territory, with the lowest annual population size (1,915,161), experienced a burst of violence in 2013, but remained relatively stable with conflict deaths not exceeding 6 in the other years.

Figure 3: Violence In North Kivu. Panel A: Annual conflict deaths and violent events between 2012-2017 in North Kivu, by territory; Panel B: Conflict Intensity by Territory; Panel c: Population estimates.

Figure 4: Trends in armed conflicts fatalities in North-Kivu between 2013 and 2018

The estimated population of South Kivu province in 2017 was 6,914,296 people. South Kivu comprises 9 territories, each of which includes 3-5 health zones (for a total of 34 in South Kivu). Between 2012 and 2017, population size varies among territories ranging from 250,000 in Idjwi island to 874,000 in Kalehe territory.

In south Kivu, the average annual numbers of violent episodes and fatalities were 15.8 and 22.4, ranging from 0.3 (in Idjwi) and 28.7 (in Uvira) and from 0 (in Idjwi) to 40.8 (Uvira) respectively. Five territories had annual average number of fatalities greater than 20, of which four had on average more than 20 events per year.

Six out of 9 territories in south Kivu had annual conflict fatality rates between 24.3 and 33.8 per 100,000 population and were classified as most affected territories in South Kivu. Three territories (Idjwi, Kalehe and Bukavu) had an average fatality rate below 9 deaths per 100,000 population and were classified as least affected territories (Figure 5). Kalehe territory also had a low conflict fatality rate, but given its high number of internal displacements, it was regarded as an intermediate territory.

The territories of Uvira and Fizi present with higher annual fatalities rates between January 2013 and June 2018 with peaks observed in 2014 and 2018 (Figure 6).

Figure 5: Violence in South Kivu. Panel a: Annual violent fatalities by territory; Panel B: Annual Average Violent Episodes; Panel C: Population Estimates; Panel D: Conflict Intensity

Figure 6: Trends in absolute number of armed conflict fatalities in South Kivu between 2012 and 2017
