# Supplementary material for: Health services for women, children and adolescents in conflict affected settings: experience from North and South Kivu, Democratic Republic of Congo
Source: Confl Health. 2020 May 27;14:31. doi: 10.1186/s13031-020-00265-1 (PMC7254646; doi:10.1186/s13031-020-00265-1)
Supplement: Supplementary file 2 — Additional file 2. Coverage estimates of selected RMNCAH+N interventions at provincial level, and related Composite Coverage Index. [file 13031_2020_265_MOESM2_ESM.docx]

Additional File 2: Coverage estimates

**RMNCH Composite Coverage Index**

Table 1: Levels and trends in composite coverage index, MICS and DHS data

| **Data source** | **BCG** | **DPT3** | **Measles** | **ORS** | **ARIHF** | **ANC1** | **SBA** | **CPR** | **FP*** | **CCI** |
| --- | --- | --- | --- | --- | --- | --- | --- | --- | --- | --- |
| **DHS 2001** |  |  |  |  |  |  |  |  |  |  |
| **Nord-Kivu** | 66.1 | 42.3 | 49.9 | 19.4 | 44.7 | 68.2 | 56.0 | 2.0 | 11.4 | 38.9 |
| **Sud-Kivu** | 42.9 | 16.7 | 35.3 | 11.6 | 27.1 | 63.6 | 56.1 | 1.5 | 9.6 | 29.2 |
| **DR Congo** | 56.4 | 30.7 | 48.0 | 16.8 | 35.8 | 68.2 | 60.7 | 4.4 | 18.1 | 37.6 |
| **DHS 2007** |  |  |  |  |  |  |  |  |  |  |
| **Nord-Kivu** | 94.5 | 83.3 | 85.8 | 46.2 | 58.1 | 94.8 | 84.9 | 13.2 | 41.6 | 67.6 |
| **Sud-Kivu** | 88 | 54.5 | 84 | 31.8 | 42.3 | 86.5 | 84 | 9.5 | 30.7 | 55.8 |
| **DR Congo** | 71.7 | 45 | 62.9 | 30.8 | 41.9 | 85.3 | 70.1 | 5.8 | 45.9 | 54.0 |
| **MICS 2010** |  |  |  |  |  |  |  |  |  |  |
| **Nord-Kivu** | 98.5 | 86.7 | 91.7 | 33.5 | 48.8 | 95.3 | 89.7 | 8.6 | 26.8 | 62.8 |
| **Sud-Kivu** | 88.3 | 54.1 | 60.5 | 23.5 | 33.4 | 87.7 | 81.9 | 5.6 | 33.8 | 52.8 |
| **DR Congo** | 84.6 | 62.1 | 72 | 26.6 | 40.3 | 87.3 | 74.9 | 5.4 | 42.3 | 56.8 |
| **DHS 2014** |  |  |  |  |  |  |  |  |  |  |
| **Nord-Kivu** | 95.1 | 87 | 85.3 | 38.7 | 29.9 | 97.4 | 91.6 | 11.6 | 30.2 | 61.9 |
| **Sud-Kivu** | 95.6 | 75.6 | 87.8 | 38.5 | 42.3 | 95.8 | 92.6 | 7.9 | 37.3 | 63.9 |
| **DR Congo** | 83.4 | 60.5 | 71.6 | 41.9 | 41.6 | 88.4 | 79.9 | 7.8 | 42.5 | 59.4 |

Family planning coverage derived from a contraceptive prevalence rate conversion formula [1].

**Coverage of selected RMNCAH+N interventions in North and South Kivu over time (available data from national surveys and DHIS2)**

Table 2: Coverage Indicators of RMNCAH+ N Interventions along the Continuum of Care, South Kivu

|  | **National value**  **(2013)** | **National surveys (DHS/MICS)** | **DHIS2** | | | | | | | | |
| --- | --- | --- | --- | --- | --- | --- | --- | --- | --- | --- | --- |
|  |  | **2001** | **2007** | **2010** | **2013** | **2012** | **2013** | **2014** | **2015** | **2016** | **2017** |
| **BCG** | 83.4 | 42.9 | 88.0 | 88.3 | 95.6 |  |  | 76.4 |  |  |  |
| **DPT 3** | 60.5 | 16.7 | 54.5 | 54.1 | 75.6 | 83.4 | 85.5 | 82.6 |  | 89.9 |  |
| **Measles** | 71.6 | 35.3 | 84.0 | 60.5 | 87.8 |  |  | 73.2 |  |  |  |
| **ORS** | 41.9 | 11.6 | 31.8 | 23.5 | 38.5 |  |  |  |  |  |  |
| **ARIHF** | 41.6 | 27.1 | 42.3 | 33.4 | 42.3 |  |  |  |  |  |  |
| **ANC1** | 88.4 | 63.6 | 86.5 | 87.7 | 95.8 | 100.4 | 99.5 | 96.2 | 88.0 | 97.7 |  |
| **SBA** | 79.9 | 56.1 | 84 | 81.9 | 92.6 | 85.46 | 82.31 | 78.91 | 80.51 | 80.07 |  |
| **CPR** | 7.8 | 1.5 | 9.5 | 5.6 | 7.9 |  |  |  |  |  |  |
| **Fpcov*** | 42.5 | 9.6 | 30.7 | 33.8 | 37.3 |  |  |  |  |  |  |
| **C-section** | 5.1 |  | 10.4 | 18.2 | 10.0 | 7.1 | 7.4 | 7.2 | 7.1 | 7.0 |  |

*Based formula conversion from the contraceptive prevalence rate

T**able 3: Prevalence of Stunting and Wasting among children under the age of 5, South Kivu**

|  | **National value**  **(2013)** | **National surveys (DHS/MICS)** | **DHIS2** | | | | | | | | |
| --- | --- | --- | --- | --- | --- | --- | --- | --- | --- | --- | --- |
|  |  | **2001** | **2007** | **2010** | **2013** | **2012** | **2013** | **2014** | **2015** | **2016** | **2017** |
| **Stunting** | 42.7 | 47.6 | 55.5 | 50.5 | 53.0 |  |  |  |  |  |  |
| **Wasting** | 7.9 | 12.2 | 7.8 | 6.0 | 7.2 |  |  |  |  |  |  |

Table 4: Coverage Indicators of RMNCAH+N Interventions along the Continuum of Care, North Kivu

|  | **National value**  **(2013)** | **National surveys (DHS/MICS)** | | | | **DHIS2** | | |
| --- | --- | --- | --- | --- | --- | --- | --- | --- |
|  |  | **2001** | **2007** | **2010** | **2013** | **2015** | **2016** | **2017** |
| **BCG** | 83.4 | 66.1 | 94.5 | 98.5 | 95.1 |  |  |  |
| **DPT 3** | 60.5 | 42.3 | 83.3 | 86.7 | 87.0 | 88.4 | 80.6 | 87.3 |
| **Measles** | 71.6 | 49.9 | 85.8 | 91.7 | 85.3 | 100.0 | 90.5 | 85.7 |
| **ORS** | 41.9 | 19.4 | 46.2 | 33.5 | 38.7 |  |  |  |
| **ARIHF** | 41.6 | 44.7 | 58.1 | 48.8 | 29.9 |  |  |  |
| **ANC1** | 88.4 | 68.2 | 94.8 | 95.3 | 97.4 | 97.0 | 92.1 | 94.2 |
| **SBA** | 79.9 | 56.0 | 84.9 | 89.7 | 91.6 | 78.5 | 72.4 | 97.8 |
| **CPR** | 7.8 | 2.0 | 13.2 | 8.6 | 11.6 | 5.7 | 5.8 | 3.5 |
| **Fpcov*** | 42.5 | 11.4 | 41.6 | 26.8 | 30.2 |  |  |  |
| **C-section** | 5.1 |  | 14.6 | 16.3 | 13.8 | 13.5 | 14.7 | 10.4 |

*Based on formula conversion from the contraceptive prevalence rate

Table 5: Prevalence of wasting and stunting among children under the age of 5, North Kivu

|  | **National value**  **(2013)** | **National surveys (DHS/MICS)** | | | |
| --- | --- | --- | --- | --- | --- |
|  |  | **2001** | **2007** | **2010** | **2013** |
| **Stunting** | 42.7 | 45.4 | 53.6 | 58.3 | 52.0 |
| **Wasting** | 13.4 | 9.9 | 6.6 | 4.5 | 4.7 |

**Figure 1: Levels and trends in MEASLES coverage in North Kivu, DHIS2 data, 2015-2017**

Measles immunization coverage in conflict settings was lower than that in stable territories and tends to decline over time.

**Figure 2: Levels and trends in ANC4 coverage in North Kivu, DHIS2 data, 2015-2017**
