# Supplementary material for: Health services for women, children and adolescents in conflict affected settings: experience from North and South Kivu, Democratic Republic of Congo
Source: Confl Health. 2020 May 27;14:31. doi: 10.1186/s13031-020-00265-1 (PMC7254646; doi:10.1186/s13031-020-00265-1)
Supplement: Supplementary file 3 — Additional file 3. Assessment of health facility data quality at health zone and provincial level (North and South Kivu). [file 13031_2020_265_MOESM3_ESM.docx]

**Assessment of health facility data quality at health zone and provincial level: North and South Kivu**

# In the wake of a widespread implementation of online health facility data collection system in many low income countries, the use of routine facility data is gaining interest in maternal and child epidemiology, including in conflict contexts [1]. Rigorous approaches have been developed to improve quality of facility data quality [2, 3] and enhance their use for generating maternal and child health statistics in low-income countries. Control checks performed on South Kivu data suggested good consistency between indicators.

# Population projections by health zone

- 1. ***Obtain the best growth rates and parameters to obtain target populations***

The census was conducted in 1984. There are however estimated population projections for the health zones available from the District Health Information System (DHIS).

- **Compute the population growth rate used in these projections.**

The multiplier being currently used by the ministry of health to estimate the population projection is 1.031. However, some health zones did have population census through mosquito nets distribution campaigns and therefore have a different growth rates for some years.

Between 2012 and 2013, the median (Q1-Q3) increase in the population size was 5.5% (3.1-11.46). Four health zones reported population increase greater than 20% in 2013 [(Kamituga (21.4%), Ibanda (36.3%), Bunyakiri (41.1%) and Kadutu (42.86%)]. In fact, these population estimates derived from mosquito nets distribution campaigns might have been biased by the inflation of the number of individuals per household/health by heads of households or health centre in-charges in order to receive more mosquito nets.

- The number of pregnancies, deliveries, births and children eligible for immunization can be computed from the total population: **summarize how this is currently done in DHIS**.
- Women of reproductive age: 21% of the total population
- Number of pregnancies: 4% of the total population
- Proportion of deliveries: number of deliveries in the health zone divided by the number of pregnant women in the health zone.
- DPT1: the denominator is set to 3.49% of the general population (proportion of children who survive)
- DHS surveys provide data on crude birth rate, as well as total fertility rates. **Assess the levels and trends, by national, urban, rural and relevant province/regions.**

|  |  | **North Kivu** | **South Kivu** | **National** | **Urban** | **Rural** |
| --- | --- | --- | --- | --- | --- | --- |
| Crude birth rate per 1,000 population | DHS 2013 |  |  | 44.1 | 40.5 | 45.9 |
|  | DHS 2007 |  |  | 44.1 | 40.4 | 46.8 |
|  |  |  |  |  |  |  |
| Total fertility rate | DHS 2013 | 6.5 | 7.7 | 6.6 | 5.4 | 7.3 |
|  | DHS 2007 | 7.0 | 7.4 | 6.3 | 5.4 | 7.0 |

- **Compare the census and survey figures**
- **Decide on the use of denominators for the districts**
- Overall population
- Population growth rate
- Crude birth rate
- **Compute (decide on multipliers)**
- Births = total population * crude birth rate (from DHS)
- Deliveries = births / 1.02 (to take account of twinning rates)
- Live births = births / 1.02
- Pregnancies for ANC1 (assuming first visit in 2^nd^ trimester): deliveries * 1.05
- Children eligible for immunization: live births * (1-neonatal mortality)

***** Neonatal mortality DHS *****

North Kivu 25/1,000 live births

South Kivu 47/ 1,000 live births

*****

**National DHIS data on DPT1 and ANC1**

The DHS 2013 showed that the coverage of ANC1 was 96-97% and DPT1 was 94-95%. The numbers of children reported in the DHIS should therefore be very close to the projected number of pregnancies and births. In addition, we expect the numbers of reported ANC and DPT to increase by at least 2.5-3% per year (the growth rate).

|  | DHS 2013 |  | DHS 2007 |  |
| --- | --- | --- | --- | --- |
|  | North Kivu | South Kivu | North Kivu | South Kivu |
| ANC1 | 97.4 | 95.8 | 94.8 | 86.5 |
| DPT1 | 94.4 | 95.1 | 93.4 | 86.2 |
|  |  |  |  |  |
| Deliveries | 91.6 | 92.6 | 84.9 | 84 |
| DPT3 | 87.0 | 75.6 | 83.3 | 54.5 |

Take into account reporting rate by health facilities

- Adjust for underreporting

Quality of reporting over time

- Compile the DPT1, ANC1 numbers by health zone and total
- Look at the trend over time: are the numbers increasing by 2-3% per year

**Annual change in ANC1 and DPT1 between 2012 and 2016**

| Year | By health zone | Total |
| --- | --- | --- |
| ANC1 |  |  |
| 2012-2013 | 4.4 (-3.1, 10.2) | 7.7 |
| 2013-2014 | -0.9 (-6.3, 5.4) | -0.3 |
| 2014-2015 | -2.6 (-8.7, 6.1) | -6.0 |
| 2014-2016 | 8.0 (-0.9, 13.5) | 11.3 |
|  |  |  |
| DPT1 |  |  |
| 2012-2013 | 6.9 (-32, 36.8) | 10.2 |
| 2013-2014 | 2.4 (-5.4, 6.7) | 1.9 |
| 2014-2015 |  |  |
| 2014-2016 | 8.2 (2.6, 15.9) | 9.5 |

DPT1 data were not available for South Kivu in 2015.

- Compute the median and the standard deviation

**Difference between expected and reported DPT1 numbers in South Kivu, from 2012 to 2016**

| Year | Median (Q1, Q2) relative difference |
| --- | --- |
| ANC1 |  |
| 2012 | 8.4 (-4.9, 16.7) |
| 2013 | 9.1 (-1.6, 18.6) |
| 2014 | 9.2 (3.1, 25.1) |
| 2015 | 14.2 (8.4, 23.9) |
| 2016 | 15.0 (8.4, 23.9) |
|  |  |
| DPT1 |  |
| 2012 | 16.73 (-19.5, 41.8) |
| 2013 | 14.7 (9.1, 18.9) |
| 2014 | 17.8 (11.5, 30.4) |
| 2016 | 13.2 (6.7, 20.5) |

DPT1 data were not available for 2015.

- Identify severe outliers (>3SD or <-3SD from median) and indicate if all numbers fall within +- 2 SD

**Outlying ANC1 and DPT1 numbers in South Kivu**

| Year | -2 and -3 SD | +2 and +3 SD | >+3 SD |
| --- | --- | --- | --- |
| ANC1 |  |  |  |
| 2012 | 1 | 1 |  |
| 2013 | 2 | 1 | 2 |
| 2014 | 1 |  |  |
| 2015 | 1 |  | 1 |
| 2016 |  | 1 |  |
|  |  |  |  |
| DPT1 |  |  |  |
| 2012 | 2 |  |  |
| 2013 |  |  |  |
| 2014 |  | 1 |  |
| 2015 |  |  |  |
| 2016 |  |  | 1 |

Data are number of health zones.

- Make any adjustments if there are obvious data entry errors (several outliers) – keep a record of the edits

Consistency between indicators

- Compare the DPT1 and ANC1 numbers – use the median of the 4-5-year period: e.g. in a scatter plot, and identify outliers.

**Figure 1. Consistency between median DPT and ANC numbers in South Kivu**

Y axis: median reported DPT1 number of 2012, 2013, 2014 and 216. X axis: median reported ANC

**Figure 2. Consistency between median reported and expected DPT1 numbers from 2012 to 2016 in South Kivu**

**Figure 3. Consistency between median reported and expected ANC1 numbers from 2012 to 2016 in South Kivu**

- The expected pattern is that DPT1 is ANC1 * (1- (pregnancy loss after 16 weeks – twin births + neonatal mortality)). In other words, .05 - .02 + (.025 or .047) = .055 or .077 smaller than ANC1.

We computed the difference between median reported numbers of ANC1 and DPT1 as (ANC1-DPT1)/ANC1 for each health zone. The median (interquartile range) ANC1-DPT1 difference was 0.13 (0.05, 0.19). In fact, 22 out 34 health zones in South Kivu had an ANC1-DPT1 relative difference greater that 0.077.

- Compare DPT3 and DPT1: according to the survey DPT3 should be about 87/94.4 =92% of the DPT1 value in North Kivu and 75.6/95.1 = 79% of the DPT1 value in South Kivu

In South Kivu, the median (IQR) and average (SD) DPT3/DPT1 ratio was 92.5% (86.7-95.9) and 91.9 %(8.8) respectively. The mean DPT3/DPT1 ratio was significantly higher than the value (79%) derived from the survey (p<0.0001). In North Kivu, the DPT3/DPT1 ratio was 95.9% (94.7-96.9), the mean (SD) was 96.6% (8.2) and significantly higher than the value (92%) estimated from the survey (p=0.0029).

Compute denominators or target population

- Obtain the estimated target population from the ANC1 and/or DPT1 data
- Compare the numbers with the population projections for a given year
- The gap between reported and expected DPT1 numbers was calculated as $\frac{\left( CEI-ANC1 \right)\times100}{CEI}$, where CEI is the number of children eligible for immunisation derived from 2013-2014 DHS estimates and DPT1 the reported DPT1 number. Similarly, the ANC1 gap was calculated as $\frac{{(ANC1}_{DHS}-{ANC1}_{DHIS2})\times100}{{ANC1}_{DHS}}$ where ${ANC1}_{DHS}$ is the estimated number of pregnancies for ANC1 and ${ANC1}_{DHIS2}$ the reported ANC1 number.

Let us compare reported and expected ANC1 and DPT1 numbers in 2014 (chosen as a median year between 2012 to 2016).

In 2014, the median (Q1-Q3) difference between expected and reported DPT1 numbers in South Kivu was 17.1% (11.5-30.4) (the mean (SD) was 18.7 %(14.7). Thirteen health zones have under-reporting rate exceeding 20%. Minova (one of the health zones where the qualitative research is going on) and Nyantende health zones had the highest over-reporting levels (9.3% and 6.4%) respectively.

**Figure 4. Consistency between reported and expected DPT1 numbers in South Kivu (2014)**

In North Kivu, the median (Q1-Q3) difference between the expected and reported DPT1 numbers was 17.0 (9.0-22.3) (the mean (SD) was 15.7 (11.5)). Fourteen (out of 33) health zones reported DPT1 numbers lower than the expected number by more than 20%, led by Oicha (one of the health zones heavily afflicted by armed conflicts) with 39% of DPT1 under-reporting. Only two health zones had higher reporting rates than expected, namely Masisi (23.1%) and Mweso (2.9%).

**Figure 5. Consistency between reported ANC1 and DPT1 numbers in North Kivu**

**Consistency between reported and expected ANC1 numbers in South Kivu (2014)**

The median (Q1-Q2) difference tween expected and reported ANC1 was 8.3% (3.1-25.1) (the mean(SD) was 11.2 (17.9). Ten health zones had under-reporting rate beyond 20% and two (Minova: 23.8% and Katana: 36.2%) of the five health zones with over-reporting had ANC1 numbers at least 20% higher than expected in South Kivu.

**Figure 6. Reported and expected ANC1 number in South Kivu in 2014**

Ibanda is the most urbanized health zone of South Kivu province located in Bukavu the capital city.

**Consistency between reported and expected ANC1 numbers in North Kivu (2017)**

The median (Q1-Q3) relative difference between expected and reported ANC1 numbers in North Kivu was 19 (10.2-28.5) and the mean (SD) was 16.8 (19.4). Fifteen of the 33 health zones of North Kivu under-reported ANC1 by more than 20%, 25 (75%) health zones under-reporting by more than 10%. Beni (50.8%), Kibirizi (41.6%) and Lubero (40.5%) were the leading health zones in terms of under-reporting. Four health zones had a reporting rate level higher than expected by 10%, 2 by 20% (Rwanguba: 32.7% and Masisi: 27%).

**Figure 7. Reported and expected ANC1 numbers in North Kivu**

**Table. Relative difference between expected and reported DPT1 and ANC1 numbers in North Kivu**

| Level | N (%) |
| --- | --- |
| DPT1 |  |
| Too low | 1 (3.0) |
| Good | 8 (24.2) |
| Too high | 18(54.6) |
| Much too high | 6(18.2) |
| ANC1 |  |
| Much too low | 2(6.1) |
| Too low | 2(6.1) |
| Good | 4(12.1) |
| Too high | 14(42.4) |
| Much too high | 8(24.2) |
| Extremely high | 3(9.1) |

Much too low: -39.5 à -25, too low: -24.9 à -9.9, good: +/-10%, too high: 10-24.9%, much too high: 25-39.9% and extremely high: ≥40.

# Reporting completeness

In South Kivu, the reporting completeness rose from its lowest level (75%) in 2013 to 96% in 2015. It peaked at 97% in 2017 after an 11% decrease in 2016 (Figure 9).

**Figure 9. Percentage of completeness of health facilities reported data over time in South Kivu**

ANC1: first antenatal care visit; DPT1: first dose of diphtheria -pertussis -tetanus vaccine; ODP: outpatient department

**ABREVIATION**

ANC1 First Antenatal Care visit

DHIS District Health Information System

DHS Demographic and Health Survey

DPT1 First dose diphtheria -pertussis -tetanus vaccine

**REFERENCES**

1. Bhattacharya AA, Umar N, Audu A, Felix H, Allen E, Schellenberg JR, Marchant T: **Quality of routine facility data for monitoring priority maternal and newborn indicators in DHIS2: A case study from Gombe State, Nigeria**. *PloS one* 2019, **14**(1):e0211265.

2. Maina I, Wanjala P, Soti D, Kipruto H, Droti B, Boerma T: **Using health-facility data to assess subnational coverage of maternal and child health indicators, Kenya**. *Bulletin of the World Health Organization* 2017, **95**(10):683.

3. Haugen JÅ, Hjemås G, Poppe O: **Manual for the DHIS2 quality tool. Understanding the basics of improving data quality**. 2017.
